# A population-based study of edentulism in the US: does depression and rural residency matter after controlling for potential confounders?

**DOI:** 10.1186/1471-2458-14-65

**Published:** 2014-01-22

**Authors:** Daniel M Saman, Andrine Lemieux, Oscar Arevalo, May Nawal Lutfiyya

**Affiliations:** 1 Essentia Institute of Rural Health, Division of Research, Duluth, MN 55805, USA; 2 Duluth Medical Research Institute, University of Minnesota Duluth, Duluth, MN 55812, USA; 3 Miami Children’s Hospital, Pediatric Dentistry, Doral, FL 33178, USA; 4 University of Minnesota, National Center for Interprofessional Practice and Education, Academic Health Center, Minneapolis, MN 55845, USA

**Keywords:** Depression, Rural residency, Edentulism

## Abstract

**Background:**

Oral health is an integral component of general health and well-being. While edentulism has been examined in relation to socioeconomic status, rural residency, chronic disease and mental health, no study that we know of has examined edentulism and these factors together. The objective of this study was to determine whether depression and rural residency were significantly associated with partial and full edentulism in US adults after controlling for potential confounders.

**Methods:**

2006 Behavioral Risk Factor Surveillance Survey (BRFSS) data were analyzed to identify factors associated with increased odds of partial or full edentulism. This year of BRFSS data was chosen for analysis because in this year the standardized and validated Personal Health Questionnaire-8 (PHQ-8) was used to measure current depression. This measure was part of the optional questions BRFSS asks, and in 2006 33 states and/or territories included them in their annual surveillance data collection. Bivariate and logistic regression analyses were performed on weighted BRFSS data.

**Results:**

Logistic regression analysis using either full or partial edentulism as the dependent variable yielded that rural residency or living in a rural locale, low and/or middle socioeconomic status (SES), depression as measured by the PHQ-8, and African American race/ethnicity were all independent risk factors when controlling for these and a number of additional covariates.

**Conclusions:**

This study adds to the epidemiological literature by assessing partial and full edentulism in the US utilizing data from the CDC’s Behavioral Risk Factor Surveillance System (BRFSS). Examining data collected through a large national surveillance system such as BRFSS allows for an analysis that incorporates an array of covariates not available from clinically-based data alone. This study demonstrated that current depression and rural residency are important factors related to partial and full edentulism after controlling for potential confounders.

## Background

Oral health is an integral component of general health and well-being [1]. In fact, former US Surgeon General C. Everett Koop noted that “You’re not healthy without good oral health” [1]. In the US, it is estimated that adults lose 164 million work hours each year due to oral health problems and dental visits [1]. Likewise, among US children, 51 million school hours are lost annually because of oral health problems, with dental caries being the most prevalent childhood disease, occurring 5 to 8 times more frequently than the second-most common condition of asthma [1]. Furthermore, research has shown that poor oral health in adults may be a risk factor associated with stroke [2], coronary heart disease [3], and acute myocardial infarction [4]. Poor oral health has also been associated with lower levels of self-esteem [5], poor mental health [6], and a lesser quality of life [7]. More specifically, edentulism has been linked with poorer quality of life among both independent-living and medically compromised elderly adults [8-10].

When the protective properties of fluoride were discovered in the 1940s leading to widespread efforts in the mid-1950s to fluoridate water and encourage the use of fluoridated toothpaste, the result was significant decreases in dental caries as well as edentulism [11-13]. For example, edentulism among persons 45–54 years of age decreased from 20% in 1960–1962 to about 9% in 1988–1994 [12]. Between 1999-2002, the Centers for Disease Control and Prevention (CDC) reported that 8% of US adults 20 years of age and older were completely edentulous [11].

Despite the inroads made with fluoridation, great disparities in oral health and oral health care still persist across multiple populations, with worse oral health outcomes found among children living in poverty [13], minorities [14], the unemployed [14], and rural residents [15]. Earlier research found that rural residents were less likely to visit a dentist because of pain and more likely to have unmet dental needs, while urban residents were more likely to have private dental insurance and to have visited a dentist within the last year [16]. Not surprisingly, a greater proportion of rural residents have been found to be edentulous and more likely to report poor dental status than urban residents [15]. Research has ascertained that lower income populations as well as rural residents often have difficulty accessing oral health care, and frequently bear significant travel burdens to access these services. The consequence has been low oral health care utilization in these populations [17-20]. The problem has been further exacerbated by the fact that many dentists do not accept Medicaid patients and Medicare excludes dental coverage [21]. Nationally, only 25% of dentists have been estimated to provide care for at least 100 Medicaid patients annually [22].

A number of researchers have noted that the stigma attached to edentulism can have a profound negative affect on mental well-being. For instance, Fiske et al., [23] found that among older edentulous patients, tooth loss was related to lowered self-confidence, dislike of appearance, altered behavior in socializing and forming close relationships, and bereavement. A recent qualitative study by Saintrain and de Souza (2012) found that among the elderly, edentulism decreased quality of life [24]. Yet another study found that the greater the number of missing teeth, regardless of age, gender, or education, the lower the levels of satisfaction with daily living [25].

Individuals living in rural communities may face multiple disadvantages associated with edentulism. For example, in addition to the difficulty in accessing oral health care as already noted, rural residents must also deal with significant deficits in mental health care regardless of the source or impetus for poor mental health [26]. Depressive symptom severity predicts lactobacillus counts independent of saliva pH, saliva flow rate, medication use (psychiatric, xerogenic and other medications), and sweet consumption [27]. This would suggest that depressive symptoms may increase an individual’s risk of poor oral health above and beyond access to dental care issues and biological covariates. Social and emotional support, however, can have a powerful buffering effect on the hypothalamic-pituitary-adrenal (HPA) activation that accompanies both stress and depression. Positive social interactions, possibly mediated by oxytocin, counteract the negative health effects of HPA activation and may account for the ability of social support to have significant effects on health [28]. Thus, while rural residents may be burdened with the multiple disadvantages of limited dental and mental health care services, not all rural residents, depressed or not, can be expected to have the same risk for edentulism.

While edentulism has been examined in relation to socioeconomic status, rural residency, chronic disease and mental health, no study that we know of has examined edentulism and these factors together. Moreover, we know of no studies on edentulism that has included the variable *health service deficits* in the tested models. This study adds to the epidemiological literature by assessing partial and full edentulism in US adults analyzing data from the CDC’s Behavioral Risk Factor Surveillance System (BRFSS). Examining data collected through a large national surveillance system such as BRFSS allows for an analysis that incorporates an array of covariates not available from clinically-based data alone. The primary objective of this study was to determine whether depression and rurality were independent risk factors for partial and full edentulism after controlling for multiple confounders such as socioeconomic status (SES), chronic disease, race/ethnicity, smoking status, and age. Moreover, we were interested in the relationship between edentulism and health service deficits.

## Methods

For this study, 2006 BRFSS data were analyzed to examine if depression and rurality were important *independent* dimensions of the epidemiology of partial and/or full edentulism while controlling for other possible confounders such as SES, health behaviors, chronic diseases, and health service deficits. The BRFSS survey is comprised of both core questions and optional modules. We chose this year of data to analyze because the optional BRFSS adult depression module was used by 33 states and/or territories. In the subsequent years of available data, many fewer states chose this option. We analyzed data collected by questions from both the core survey as well as the optional adult depression module based on the Personal Health Questionnaire-8 (PHQ-8).

BRFFS data are collected using a random-digit dial telephone survey targeting adults 18 through 99 years of age. These data are collected under the guidance of the CDC in collaboration with all US states and most US territories. Once collected, the data are weighted by state or territory to represent the non-institutionalized US adult population based on the most recent census data available. BRFSS data are cross-sectional and are focused on health risk factors and behaviors as well as chronic disease. A detailed description of the survey design and sampling measures can be found elsewhere [29].

In the analyses presented here a number of variables were either re-coded or computed. Re-coding for the most part entailed collapsing response categories and removing the response categories of don’t know and refused. The following variables were computed: chronic disease index, health service deficits, socioeconomic status and current depression.

Chronic disease index (CDI) entailed combining the variables of diabetes and cardiovascular disease. Anyone having one or both of the diseases was categorized as having at least one chronic condition related to edentulism.

Health service deficits, one of the independent variables in this analysis, was computed from the response categories of four separate variables (health insurance status, personal healthcare provider, deferment of medical care because of cost, routine medical exam). Health service deficits is a proxy for health care coverage and utilization since the BRFSS asks no questions about dental insurance. The response categories included in the computation of the variable were: did not have health insurance, did not have a healthcare provider, deferred medical care because of cost, and did not have a routine medical exam, all within the last 12 months. Together these four issues form a constellation of factors that can and often lead to deficits in care in the US health system. These four issues are interwoven and since health service deficits is an evolving concept they are given equal weight in this analysis. Having at least one of these constituted having a health service deficit.

SES was also one of the primary independent variables. SES is one of the strongest determinants of health [30]. While it is a commonly used term in analyses across disciplines (e.g., sociology, social epidemiology, social psychology), there is no general consensus about how to either define or measure the construct [31-33]. Typically SES refers to a combination of household income and other social measures such as attained educational level indexed into a single variable [31]. The purpose of SES is to provide some means of comparing relative position with regard to others. Almost always, SES is computed as a three-level variable (i.e., low, middle and high) [33]. Various measures of SES are typically not interchangeable and reflect the intent and approach of the investigator [33]. In our analyses, SES was a computed variable comprised of two categorical variables: attained education and median annual household income. In keeping with convention, data categories from each of these individual variables were coded as one of low, mid-range or high and numbered 1, 2 or 3 respectively. The variables with numbered factors or categories were then added together to create the composite variable of SES. For education, low was less than high school and was coded as 1, mid-range was high school graduate and was coded as 2, and high was at least some college and was coded as 3. For income, low referred to the category < $25,000 and was coded as 1, mid-range referred to $25,000 - < $50,000 and was coded as 2, and high equaled ≥ $50,000 and was coded as 3. When the individual variables were added together the possible computed range was 2–6 points. These points were then indexed in the following manner: low = 2–3 points, mid-range = 4–5 points and high = 6 points. These cut-points were purposive. For the lowest range of the index, 2 points were the floor (smallest possible point assignment), for the mid-range of the index, 4 points was the floor and likewise for the high range of the index, 6 points was the floor. Any points below the floor for the mid-range were assigned to the lowest index category just as any points below the floor for the highest index category were assigned to the mid-range index category.

The standardized and validated PHQ-8 was used to measure current depression. This validated instrument consists of eight of the nine criteria on which the Diagnostic and Statistical Manual of Mental Disorders 4^th^ Edition Revised Text (DSM-IV-TR) diagnosis of depressive disorders is based [34]. The ninth question in the DSM-IV-TR assesses suicidal or self-injurious thoughts. It is omitted because interviewers/researchers were not able to provide adequate intervention by telephone if a respondent indicates that they were having such thoughts [35]. The PHQ-8 response set was standardized to make it similar to other BRFSS questions by asking the number of days in the past two weeks the respondent had experienced a particular depressive symptom. Similar to a methodology employed by other researchers [35,36], the modified response set was converted back to the original response set: 0 to 1 day = *not at all*, 2 to 6 days = *several days*, 7 to11 days = *more than half the days*, and 12 to 14 days = *nearly every day*, with points (0 to 3) assigned to each category, respectively. The scores for each item were summed to produce a total score between 0 and 24 points. A total score of 0 to 4 represents no significant depressive symptoms. A total score of 5 to 9 represents mild depressive symptoms; 10 to 14, moderate; 15 to 19, moderately severe; and 20 to 24, severe. This is summarized in Table 1. For our analyses, current depression was defined as: a PHQ-8 score of ≥ 10, which has 88% sensitivity and 88% specificity for major depression and, regardless of diagnostic status, typically represents clinically significant depression [35,36].

**Table 1:** **Patient health questionnaire (PHQ-8) scoring and interpretation with BRFSS response conversion **[17]

**Over the *last 2 weeks, *how often have you been bothered by any of the following problems?**	**PHQ-8**	**Not at all**	**Several days**	**More than half the days**	**Nearly every day**
	**BFRSS conversion**	**0 - 1 day**	**2 - 6 days**	**7 - 11 days**	**12 - 14 days**
**1.** Little interest or pleasure in doing things		0	1	2	3
**2.** Feeling down, depressed, or hopeless		0	1	2	3
**3.** Trouble falling or staying asleep, or sleeping too much		0	1	2	3
**4.** Feeling tired or having little energy		0	1	2	3
**5.** Poor appetite or overeating		0	1	2	3
**6.** Feeling bad about yourself—or that you are a failure or have let yourself or your family down		0	1	2	3
**7.** Trouble concentrating on things, such as reading the newspaper or watching television		0	1	2	3
**8.** Moving or speaking so slowly that other people could have noticed. Or the opposite—being so fidgety or restless that you have been moving around a lot more than usual		0	1	2	3

Interpretation of Total Score/Total Score Depression Severity: 0–4 None, 5–9 Mild depression, 10–14 Moderate depression, 15–19 moderately severe depression, 20–24 severe depression.

The Metropolitan Statistical Area (MSA) variable included in BRFSS was used to define place of residence as either rural or non-rural. Rural residents were defined as persons living either within an MSA that had no city center or outside an MSA. Non-rural residents included all respondents living in a city center of an MSA, outside the city center of an MSA but inside the county containing the city center, or inside a suburban county of the MSA.

Race and ethnicity was calculated from participant responses to two separate survey questions—one regarding race and the other regarding Latino/Hispanic ethnicity. All race/ethnicity categories were computed as *mutually exclusive* entities. For example, all respondents coded as Caucasian chose white as their racial classification, likewise, black for African American, etc. If a respondent identified themselves as Hispanic or Latino they were classified by that ethnic category regardless of any additional racial classification. The category of Other/Multiracial was also calculated.

Data analyses entailed both bivariate and multivariate techniques. Our population of interest was non-institutionalized adults completing the PHQ-8 depression screening tool as part of the 2006 BRFSS survey. Two logistic regression models were performed, one using partial edentulism as the dependent variable and the second using full edentulism as the dependent variable. SPSS (IBM, Chicago, Illinois) version 21.0 was used for all of the analyses. Alpha was set a p > =.05. This was a database study; as such human subjects’ approval was not necessary.

## Results

Table 2 describes the study population. These data revealed that according to the PHQ-8, 9.2% of the population was depressed or exhibited depressive symptoms. Additionally, 14.0% had at least one chronic condition, 42.4% had at least one health service deficit, 27.3% were classified as being low SES, 21.3% were rural residents, and 18.6% were current smokers. In addition, 14.3% had all of their teeth removed (full edentulism) while 48.8% were partially edentulous.

**Table 2:** **Description of study population 2006 BRFSS (weighted n = 122671734**)

**Variable**	**Factor**	**% of study population**
Gender	Male	37.7
	Female	62.3
Self-Defined Health Status	Fair To Poor	18.4
	Good To Excellent	81.6
Heavy Drinker	Heavy Drinker	4.7
	Not A Heavy Drinker	95.3
Smoking Status	Current Smoker	18.6
	Non-Smoker	81.4
Age	<65 Years	74.8
	> = 65 Years	25.2
Race/Ethnicity	Caucasian	77.4
	African American	8.4
	Hispanic	9.2
	Other/Multiracial	5.0
Marital Status	Not Married/Living With Partner	42.5
	Married/ Living With Partner	57.5
Children At Home	No Child At Home	66.4
	At Least One Child At Home	33.6
Employment	Employed	55.8
	Unemployed	4.2
	Not Working By Choice	33.9
	Unable To Work	6.1
Feel Emotionally Supported	Rarely To Never	8.8
	Sometimes To Always	91.2
Geographic Locale	Rural	21.3
	Non-Rural	78.7
Health Service Deficits	At Least One HSD	42.4
	No HSD	57.6
Socioeconomic Status	Low	27.3
	Mid-Range	48.3
	High	24.4
Phq-8 Depression	Currently Depressed	9.2
	Not Currently Depressed	90.8
Chronic Disease Index	At Least One Chronic Disease	14.0
	No Chronic Disease	86.0
Edentulism	All Teeth Removed	14.3
	Partial Teeth Removed	48.8
	No Teeth Removed	36.9

A bivariate analysis (Table 3) examining full edentulism by study covariates yielded that rural residents, current smokers, those not living with a partner, those without children living at home, those expressing that they have little emotional support, those who were exhibiting depression symptoms according to the PHQ-8, and those rating their health status as good to excellent had greater odds of full edentulism. In addition 64% were of lower SES and 60.0% reported not working by choice. US adults who reported full edentulism had lesser odds of having a health service deficit.

**Table 3:** Bivariate analysis of US adults with full edentulism 2006 BRFSS data (weighted n = 66357961)

**Variables and factors**		**Unadjusted odds ratio(95% CI)**
Gender (vs. Female)	Male	.848 (.847, .849)
Geographic Locale (vs. Non-Rural)	Rural	1.876 (1.874, 1.878)
Self-defined Health Status (vs. Fair to Poor)	Good to Excellent Health	4.515 (4.510,4.520)
Heavy Drinker (vs. Not a Heavy Drinker)	Heavy Drinker	.512 (.510, .514)
Smoking Status (vs. Non-Smoker)	Current Smoker	2.000 (1.997, 2.002)
Age (vs. 65 and Older)	18-64 Years	.136 (.136, .136)
Marital Status (vs. Married or Living with Partner)	Not Married or Living With Partner	2.336 (2.333, 2.339)
Children (vs. at least one child living at home)	No Children living at home	5.933 (5.921, 5.945)
Feel Emotionally Supported (vs. sometimes to always)	Rarely to never	2.340 (2.337, 2.344)
Health Service Deficits (vs. No Health Service Deficits)	At Least One Health Service Deficit	.682 (.682, .683)
PHQ-8 Score (vs. Not Depressed)	Depressed	2.167 (2.162, 2.172)
**Variables and factors**		**% of adults with full edentulism**
Race and Ethnicity (% by Race)	Caucasian	80.2
	African American	9.8
	Hispanic	5.3
	Other/Multiracial	4.8
Employment Status	Employed	20.6
	Unemployed	3.2
	Not Working By Choice	60.0
	Unable to Work	16.2
Socioeconomic Status	Low	64.6
	Mid-Range	32.4
	High	3.0

Table 4 displays a bivariate analysis examining partial edentulism by all of the study covariates. Rural residents, those self-defining their health as good to excellent, current smokers, those with no children living at home, those expressing a lack of emotional support, and those experiencing depressive symptoms as measured by the PHQ-8 had greater odds of partial edentulism. US adults who reported partial edentulism had lesser odds of having a health service deficit.

**Table 4:** Bivariate analysis of US adults with partial edentulism 2006 BRFSS data (weighted n = 66357961)

**Variables and factors**		**Unadjusted odds ratio (95% CI)**
Gender (vs. Female)	Male	.997 (.997, .998)
Geographic Locale (vs. Non-Rural)	Rural	1.167 (1.167, 1.168)
Self-defined Health Status (vs. Fair to Poor)	Good to Excellent Health	1.555 (1.554, 1.555)
Heavy Drinker (vs. Not a Heavy Drinker)	Heavy Drinker	.891 (.890, .892)
Smoking Status (vs. Non-Smoker)	Current Smoker	1.278 (1.277, 1.278)
Age (vs. 65 and Older)	18-64 Years	.586 (.585, .586)
Marital Status (vs. Married or Living with Partner)	Not Married or Living With Partner	1.193 (1.193, 1.194)
Children (vs. at least one child living at home)	No Children living at home	1.539 (1.538, 1.539)
Feel Emotionally Supported (vs. sometimes to always)	Rarely to never	1.307 (1.306, 1.308)
Health Service Deficits (vs. No Health Service Deficits)	At Least One Health Service Deficit	.926 (.926, .927)
PHQ-8 Score (vs. Not Depressed)	Depressed	1.361 (1.360, 1.362)
**Variables and factors**			**% of adults with partial edentulism**
Race and Ethnicity (% by Race)	Caucasian	74.8%	
	African American	10.6%	
	Hispanic	9.5%	
	Other/Multiracial	5.2%	
Employment Status	Employed	49.5%	
	Unemployed	4.7%	
	Not Working By Choice	37.9%	
	Unable to Work	7.9%	
Socioeconomic Status	Low	33.5%	
	Mid-Range	50.4%	
	High	16.2%	

Table 5 displays a bivariate analysis of the US adult population by edentulism status (full or partial) and geographic locale by SES, health service deficits, smoking status, and PHQ-8 depression. Analysis revealed that both rural and non-rural adults by edentulism status exhibited an SES gradient with a greater proportion of adults in the low SES group and a smaller proportion in the high SES group. The gradient was steeper for rural adults by edentulism status. Disparities were revealed for rural adults for health service deficits and smoking status (current smoker) for both edentulism groups. PHQ-8 depression was not significantly different for any of the groups.

**Table 5:** Bivariate analysis of US adult population by edentulism status (full or partial) and geographic locale by SES, health service deficits, smoking status, and PHQ-8 depression (% or unadjusted odds ratio) 2006 BRFSS data

**Covariates and factors**		**Full (n = 9418006)**		**Partial (n = 54019019)**	
		**Rural**	**Non-rural**	**Rural**	**Non-rural**
SES	Low	70.8%	61.7%	39.6%	31.6%
	Mid-Range	27.6%	34.6%	50.1%	50.4%
	High	1.5%	3.7%	10.3%	18.0%
Health Service Deficits	At Least One HSD	1.154 (1.152, 1.157)	.933 (.933, .934)	1.119 (1.118, 1.120)	.967 (.966, .967)
Smoking Status	Current Smoker	1.070 (1.067, 1.072)	.969 (.968, .969)	1.115 (1.114, 1.117)	.967 (.966, .967)
PHQ-8 Depression	Currently Depressed	.996 (.993, 1.000)	1.002 (1.000, 1.004)	1.050 (1.048, 1.052)	.985 (.984, .986)

Two logistic regression models were performed and the results are displayed in Table 6. Logistic regression analysis using either full or partial edentulism as the dependent variable yielded that rural residency or living in a rural locale, low and/or middle SES, depression as measured by the PHQ-8, and African American race/ethnicity were all independent risk factors when controlling for these and a number of additional covariates. In addition, greater odds of either full or partial edentulism were associated with: being unable to work, self-defined health status as fair to poor, being a smoker, being ≥ 65 years of age, having at least one related chronic disease, and rarely to never feeling emotionally supported.

**Table 6:** Logistic regression analysis full or partial edentulism 2006 BRFSS data

** Variables**	** Factors**	**Adjusted odds ratios (95% CI)**	
		**Full**	**Partial**
Employment	Employed	-*	-*
	Unemployed	1.059(1.051,1.066)	1.196 (1.192,1.200)
	Not Working By Choice	1.511(1.505,1.516)	.992 (.990,.993)
	Unable To Work	2.597(2.583,2.612)	1.505 (1.500,1.510)
Race/Ethnicity	Caucasian	-*	-*
	African American	1.454(1.446,1.462)	2.158 (2.153,2.163)
	Hispanic	.525(.522,.528)	1.063 (1.060,1.065)
	Other/Multiracial	1.055(1.049,1.062)	1.341 (1.338,1.345)
Socioeconomic Status	Low	15.913(15.812,16.015)	3.120 (3.113,3.126)
	Mid-Range	4.650(4.622,4.679)	1.935 (1.932,1.938)
	High	-*	-*
PHQ-8 Depression	Currently Depressed	1.212(1.206,1.219)	1.315 (1.311,1.318)
	Not Currently Depressed	-*	-*
Self-Defined Health Status	Fair To Poor	1.919(1.912,1.926)	1.480 (1.477,1.483)
	Good To Excellent	-*	-*
Heavy Drinker	Heavy Drinker	.594(.590,.598)	.830 (.827,.832)
	Not A Heavy Drinker	-*	-*
Smoking Status	Current Smoker	3.592(3.579,3.604)	1.871 (1.868,1.874)
	Non-Smoker	-*	-*
Age For Analysis	<65 Years	-*	-*
	> = 65 Years	6.535(6.509,6.561)	3.029 (3.023,3.035)
Marital Recoded	Not Married/Living With Partner	.860(.858,.863)	.792 (.790, .793)
	Married/ Living With Partner	-*	-*
Children At Home	No Child at Home	-*	-*
	At Least One Child At Home	.379(.378,.381)	.625 (.624,.626)
Chronic Disease Index	At Least One Chronic Disease	2.404(2.395,2.413)	1.644 (1.641, 1.648)
	No Chronic Disease	-*	-*
Health Service Deficits	At Least One HSD	.932(.929,.935)	.934 (.933,.936)
	No HSD	-*	-*
Geographic Locale	Rural	1.627(1.621,1.632)	1.226 (1.224,1.228)
	Non-Rural	-*	-*
Feel Supported Emotionally	Rarely To Never	1.261(1.255,1.268)	1.202 (1.199,1.205)
	Sometimes To Always	-*	-*
Gender	Male	1.058(1.055,1.061)	1.048 (1.046,1.049)
	Female	-*	-*

*Reference Category.

## Discussion

Descriptive analysis revealed that 14.3% of US adults had all of their teeth removed, while 48.8% were partially edentulous. Given our research question, the most significant findings were that after controlling for multiple risk factors including SES, race/ethnicity, and chronic disease---rural residency and depression (as measured by PHQ-8) were independent risk factors for both partial and full edentulism. Adults with full edentulism were 62.7% more likely to be rural and 21.2% depressed. Likewise, adults who were partially edentulous were 22.6% more likely to be rural residents and 31.5% depressed. For a number of reasons these findings are important.

First, a critical initial step to addressing health and health care disparities including edentulism is an appreciation and identification of who may be at risk [37]. This notion is grounded in the understanding that societies shape patterns of disease and that these patterns change over time in response to multiple factors [38,39]. A growing body of literature indicates the complex social and economic interaction geographic locale contributes to health disparities independent of many individual-level risk factors [40]. While disparities in health and health care among minorities and those of low SES are a well-recognized problem, rural health disparities is becoming more recognized [41-43] and suggests that rural culture may be a health determinant [41,44]. Our findings add to this growing body of knowledge establishing rural residency as a risk factor and heightens an appreciation of the need to develop strategies that incorporate geography into programs that target the management of essential health issues such as edentulism.

Second, the association between depression and both partial and full edentulism underscores the connection of mental health with physical ailments and conditions. Because this study was cross-sectional it is not possible to determine a causal link between edentulism and depression. Moreover while associations between lower levels of self-esteem [5], poor mental health [6], and a lesser quality of life [7] have been established in other studies, this study found an association between edentulism and depression with the latter established through a validated measure (PHQ-8). The findings from at least one other study revealed that tooth loss was associated with depression and anxiety after controlling for multiple confounders [45]. Despite the lack of a causal relationship, it is not implausible to suggest that oral health care providers screen patients facing some level of tooth loss for depression. Likewise, health care providers in other settings who detect depression or anxiety in patients should query the patient about their oral health.

Third, understanding the impact of health service deficits is also important because it provides an indication how many individuals may or may not be receiving medical care. This study found that US adults who reported either full or partial edentulism had lesser odds of having a health service deficit. As defined in this study, health service deficits are tied to medical care in the past twelve months (not having health insurance, not having a health care provider, deferring care because of cost and not having a routine physical exam) of which oral health care is not a part. Our findings suggest that edentulous (partial or full) adults did not experience a health service deficit. In many ways this finding speaks to the way dental health is financed in the US. Even with dental health insurance those seeking oral health care still have considerable out of pocket costs and this may very well influence oral health care decisions. With medical health insurance, out of pocket expenses are typically not as steep. This finding dovetails with the finding regarding SES---that edentulous adults are more likely to be either of low SES or mid-range SES. A recent paper by Manski, et al., [46] found that the likelihood of accessing dental care decreases with a decline in income as well as wealth.

Since rural residents are at greater risk for edentulism, policies targeted toward these individuals might be beneficial. In addition to providing better access to clinicians, programs that improve rural residents’ access to nutrition counseling, oral hygiene, and oral health care providers, might prove beneficial as well.

### Limitations

Several potential limitations to this study should be noted. First, the survey is based on telephone interview derived data and may be skewed because those who could not be reached by phone could not participate in the survey. For example, the wide-spread use of answering machines and caller ID allow people to filter their phone calls potentially leading to a passive refusal to participate in health surveillance surveys such as the BRFSS. However, the use of answering machines and caller ID to filter out “unwanted” or “unfamiliar” callers is beyond the control of survey administrators. In addition, some persons of lower SES may have been excluded because of poorer phone access, but the fact that the vast majority of US residents live in households with telephones minimize this bias. Furthermore, US cell phone numbers are now included in the pool of phones contacted for the survey.

A second limitation is that the survey used close-ended questions, which limit responder’s options to fully explain response choices. However, the survey questions were worded such that the answer choices covered a wide range of response possibilities. A third and related limitation is that the answers are self-reported, which introduces the possibility of exposure and outcome misclassification on the part of the survey participants.

A fourth limitation is that only those variables available from the survey questions could be used and these questions may not reflect a fully comprehensive measure of the concepts included in the analyses. Finally, this study analyzed cross-sectional data, limiting assessment of causal relationships. At best associations are detectable in cross-sectional studies such as the one presented here. Furthermore, at this point we are uncertain as to whether identified associations are differential with respect to individual components of the computed variables. Further analysis will examine those associations.

## Conclusions

This study adds to the epidemiological literature by assessing partial and full edentulism in the US utilizing data from the CDC’s Behavioral Risk Factor Surveillance System (BRFSS). Examining data collected through a large national surveillance system such as BRFSS allows for an analysis that incorporates an array of covariates not available from clinically-based data alone. This study demonstrated that current depression and rural residency are important factors related to partial and full edentulism after controlling for potential confounders.

## Competing interests

The authors declare that they have no competing interests.

## Authors’ contributions

DMS, AL, OA, and MNL all made substantial contributions to the conception and design of the manuscript, contributed to the interpretation of the data, were involved in revising the manuscript critically for important intellectual content, and have given final approval of this version of the manuscript to be published. Additionally, MNL oversaw the statistical analyses and the acquisition of the data. All authors read and approved the final manuscript.

## Pre-publication history

The pre-publication history for this paper can be accessed here:

http://www.biomedcentral.com/1471-2458/14/65/prepub
